# A study on the emotional accuracy of generative AI music from the perspective of the “Emotion Wheel” theory

**DOI:** 10.3389/fpsyg.2026.1794715

**Published:** 2026-07-17

**Authors:** Gao Qi, Dong Song, Xia Yu

**Affiliations:** 1 School of Music and Dance, Huaihua University, Huaihua, China; 2 Global Convergence Studies Department, Kangwon National University, Chuncheon-si, Republic of Korea

**Keywords:** emotional expression accuracy, generative AI music, musical emotion, Suno AI, Wheel of Emotions theory

## Abstract

**Introduction:**

In the contemporary landscape of information technology, the advent of artificial intelligence (AI) in music composition marks a transformative era. This study examines differences in emotional expression accuracy between AI-generated music and compositions by traditional musicians, with a particular focus on Suno AI within the theoretical framework of Plutchik’s “Wheel of Emotions.” Emotional expression accuracy is defined as the degree of correspondence between the intended target emotion of a musical piece and the emotion perceived by listeners.

**Methods:**

An empirical approach was adopted to compare AI-generated and human-composed music and to evaluate the accuracy of emotional expression across four primary high-intensity emotions (ecstasy, rage, grief, and terror). A total of 32 musical pieces were analyzed, comprising 16 compositions by traditional composers and 16 works generated by Suno AI. Survey data were collected from 300 university students without formal music training, yielding 283 valid responses.

**Results:**

The results revealed that emotional expression accuracy differed significantly between human-composed and AI-generated music for rage (χ^2^ = 40.66, *p* < 0.001, Cramer’s *V* = 0.27), ecstasy (χ^2^ = 25.53, *p* < 0.001, *V* = 0.21), and terror (χ^2^ = 16.46, *p* < 0.001, *V* = 0.17), whereas no significant difference was observed for grief (χ^2^ = 2.44, *p* = 0.12, *V* = 0.07). Across emotions, the largest accuracy gap was observed for rage (25.40%), followed by ecstasy (21.50%) and terror (17.00%), while the difference for grief was comparatively smaller (4.20%) and not statistically significant.

**Discussion:**

These findings suggest that the advantage of human-composed music over AI-generated music may vary across emotional categories, with more pronounced differences observed for high-arousal emotions. The results further indicate that, despite substantial advances in generative systems such as Suno AI, current AI models may still face limitations in representing high-intensity emotional states. Future research could further explore improvements in emotional conditioning mechanisms and training data diversity to enhance the emotional expression accuracy of AI-generated music.

## 1 Introduction

The integration of artificial intelligence (AI) into various sectors has not spared the music composition industry. Since the early 21st century, advances in computational power and algorithms have promoted the development of generative AI music systems based on deep learning and neural networks. Unlike earlier simple rule-based systems, contemporary AI music technologies now harness sophisticated models such as Recurrent Neural Networks (RNNs), Generative Adversarial Networks (GANs), and Variational Autoencoders (VAEs). These models facilitate the creation of compositions that are not only musically varied but also structurally intricate (Zhang, 2025; Wei et al., 2025).

Illustrative of these advancements are Google’s Magenta project and OpenAI’s MuseNet. Magenta’s NSynth employs deep neural networks to synthesize finely detailed and lifelike musical tones, while MuseNet, utilizing Transformer architecture, is adept at producing music that spans genres from classical to contemporary. These developments underscore the profound potential of AI in enhancing musical creativity (Wang and Zhang, 2025).

Despite these technological advances, the investigation of emotional processes in AI-generated music remains limited. Music, as an expressive art form, involves not only the encoding of emotional intent by the composer but also the decoding of emotional meaning by listeners. In this study, emotional expression accuracy refers to the degree to which a musical piece successfully conveys its intended emotion, operationalized as the correspondence between the pre-assigned target emotion of a composition and the emotion perceived by listeners (Juslin and Timmers, 2010). In contrast, emotional perception accuracy refers to the listener’s ability to correctly identify or interpret the emotional content of a given musical stimulus (Eerola and Vuoskoski, 2013). This study focuses on expression accuracy as reflected through perceptual agreement, thereby providing a more precise operational framework for evaluating emotional expression accuracy in music.

Despite growing interest in AI-generated music, empirical studies examining its emotional expressiveness remain relatively scarce (Jiang et al., 2024). Therefore, understanding and delineating the emotional expressiveness of generative AI music vis-à-vis traditional composers is not only of academic interest but also of practical significance (Gao et al., 2025). This study aims to examine differences in emotional expression accuracy between music generated by Suno AI and human-composed works, thereby identifying both the capabilities and the limitations of current AI systems in conveying affective content (Ma and Zheng, 2025).

## 2 Theoretical foundation and literature review

Generative AI music technology has evolved over 70 years since the 1950s. Robert Plutchik’s Wheel of Emotions Theory has also been employed in various research domains. However, utilizing the “Plutchik’s Wheel of Emotions” to study the affective aspects of generative AI music technology represents a novel endeavor.

### 2.1 Development trajectory of generative AI music technology

Generative AI music technology has undergone significant evolution since its inception in the 1950s. The integration of computer science with music theory marked the beginning of this field. One of the earliest milestones was the creation of the Illiac Suite by Lejaren Hiller and Leonard Isaacson at the University of Illinois in 1957, recognized as the first piece of computer-generated music. This pioneering work employed Markov chains and stochastic processes to compose music. Throughout the 1960s and 1970s, the focus predominantly lay on rule-based and knowledge-driven systems which utilized predefined musical rules to generate compositions. David Cope’s Experiments in Musical Intelligence (EMI) is a notable example from this era, analyzing existing musical works to extract styles and rules for new compositions (Cope, 1989). In the 1980s and 1990s, advances in computing and neural network techniques introduced a new stage in AI music development. These developments allowed for the application of machine learning algorithms in music generation (Pricop and Iftene, 2024).

The rise of deep learning technologies in the 21st century introduced profound changes, with Recurrent Neural Networks (RNNs) and Long Short-Term Memory networks (LSTMs) proving particularly effective for handling time-series data pertinent to music generation (Ge, 2024).

Recent innovations include the use of Generative Adversarial Networks (GANs) and Variational Autoencoders (VAEs) to generate more realistic and complex music. For instance, OpenAI’s MuseNet in 2017 and Google’s Magenta project with its NSynth system have showcased the versatility and potential of these technologies. In 2024, an AI startup in Cambridge, Massachusetts, introduced the Suno AI V3 music model, capable of generating 2 min of audio in mere seconds, indicating a promising direction for future developments in AI music technology.

However, despite these technological developments, the question of emotional expressiveness in AI-generated music remains insufficiently explored. While early research primarily emphasized structural coherence and stylistic imitation, recent studies in music psychology have increasingly shifted attention toward how effectively AI-generated music conveys and evokes emotions in listeners. For instance, recent studies have shown that although participants tended to perceive human-composed music as more emotionally effective, no significant differences were found in actual emotional responses. Interestingly, findings regarding listener preferences remain mixed, the perceptions of authenticity and source attribution may play a role in shaping evaluations of AI-generated music (Lecamwasam and Chaudhuri, 2025; Chia et al., 2025; Xiao, 2025).

More importantly, recent neuroscience-oriented studies have further highlighted the emotional regulation potential of AI-generated music. Using an EEG-based framework combined with convolutional neural networks (CNNs), researchers found that AI-generated personalized music significantly increased alpha-band activity while reducing beta and gamma activity, indicating a shift toward relaxation. The system achieved over 90% emotion-classification accuracy and was associated with reduced stress and enhanced wellbeing, suggesting that AI-generated music may have practical value in emotion regulation and affective computing contexts (Martin et al., 2025).

Therefore, understanding and systematically evaluating the emotional expressiveness of generative AI music has become an important research direction. This research is important for both theoretical studies of music cognition and practical applications in media, entertainment, and therapy.

### 2.2 Robert Plutchik’s Wheel of Emotions theory

**FIGURE 1:**
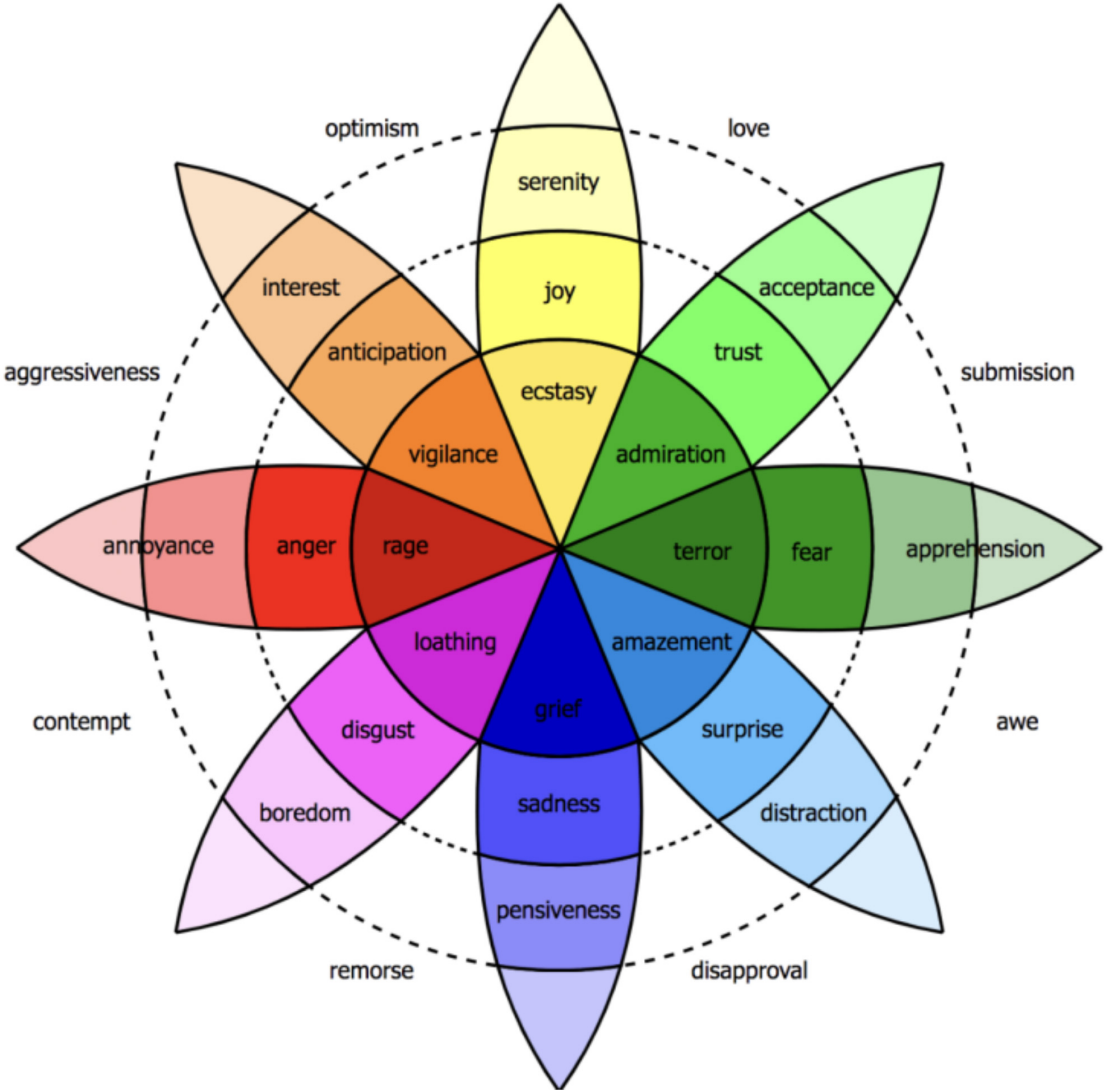
Robert Plutchik’s Wheel of Emotions theory. Source: https://zh.wikipedia.org/wiki/%E7%BE%85%E4%BC%AF%E7%89%B9%C2%B7%E6%99%AE%E6%8B%89%E5%A5%87%E5%85%8B.

Robert Plutchik’s “Wheel of Emotions” theory is a seminal psychological framework designed to categorize and explain basic human emotions. According to Plutchik, emotions are subjective responses to experiences and are interconnected in a complex spectrum akin to colors (Semeraro et al., 2021). The structure of Plutchik’s Wheel of Emotions is illustrated in Figure 1. The wheel comprises eight primary emotion groups, each centered around a core emotion. These include ecstasy, admiration, terror, amazement, grief, loathing, rage, and vigilance. Each group displays varying intensities spread across three concentric circles, with more intense emotions at the center and milder ones toward the outer edges.

The theory posits that emotions within the same group share substantial similarities, while those from different groups exhibit pronounced differences. Emotions diametrically opposite on the wheel, such as ecstasy and grief, represent the most substantial contrasts. This nuanced arrangement allows for the combination of primary emotions to form complex emotional states, for example, optimism results from the combination of ecstasy and anticipation, and love emerges from ecstasy and trust (Mohsin and Beltiukov, 2019; Qi et al., 2019; Warpechowski et al., 2019).

In Plutchik’s model, the eight primary emotions are structurally organized into four pairs of polar opposites: ecstasy versus grief, admiration versus loathing, terror versus rage, and amazement versus vigilance. This dimensional structure provides a theoretical foundation for investigating emotional expression, as polar-opposite pairs represent the most emotionally distinct and least ambiguous categories, thereby facilitating clearer differentiation by listeners. In this study, four emotions including ecstasy, rage, grief, and terror were selected from two opposing pairs (ecstasy vs. grief and rage vs. terror).

This selection strategy was theoretically motivated by two considerations. First, focusing on polar-opposite pairs maximizes emotional contrast, reduces the risk of perceptual confusion, and enhances the reliability of listener judgments in forced-choice tasks. Second, these four emotions are widely recognized in music psychology research as core affective states that can be reliably conveyed through instrumental music without the aid of lyrical content, thereby ensuring the validity of cross-category comparisons. The remaining four emotions (admiration, loathing, amazement, and vigilance) were excluded to maintain a controlled experimental design with clearly distinguishable target emotions, reduce participant fatigue and cognitive load, and preserve sufficient statistical power.

This framework provides a basis for examining whether AI-generated music can effectively convey complex emotional expressions.

In the present study, Plutchik’s Wheel of Emotions was adopted as the theoretical framework for emotion classification and stimulus evaluation. The current study did not operationalize the eight core emotions located in the middle layer of the wheel (e.g., joy, sadness, anger, and fear). Instead, it employed the high-intensity variants positioned in the outer layer of the model—ecstasy, grief, rage, terror, admiration, amazement, loathing, and vigilance—as discrete response categories. This distinction is important because Plutchik conceptualized emotions as intensity gradients rather than isolated categories, with outer-layer emotions representing amplified forms of corresponding core emotions (Semeraro et al., 2021).

Compared with moderate emotional states, high-arousal emotions are generally associated with clearer acoustic cues, including tempo variation, dynamic contrast, timbral intensity, and harmonic instability, making them more suitable for experimental research on music emotion perception. Using these eight categories allowed the study to establish a standardized forced-choice recognition task and measure emotional recognition accuracy as the correspondence between intended and perceived emotions.

Beyond its role as an experimental framework, the Emotion Wheel also demonstrates broad applicability in domains related to emotional identification and regulation. In mental health and therapy, it facilitates the accurate identification and expression of emotions, which is crucial for managing affective disorders such as anxiety and depression (Mukhopadhyay et al., 2020). In educational contexts, it supports the development of emotional awareness and regulation skills, thereby enhancing learning efficiency and social-emotional competence. In the field of artificial intelligence and machine learning, the model has been applied to the development of emotion recognition systems, enabling more nuanced interpretation and response to human emotional states (Butkute, 2023).

These applications highlight the theoretical robustness and practical versatility of the Emotion Wheel, underscoring its value not only as a psychological model but also as a foundational framework for interdisciplinary research spanning education, technology, and human-centered design (Agarwal, 2022; Abukhodair et al., 2024; Liu, 2023).

### 2.3 Research status and literature review

Music elicits profound emotions such as happiness, grief, rage, fear, excitement, and relaxation through melodic, rhythmic, and harmonic structures. Different musical elements can induce specific emotional states; for example, cheerful rhythms and major melodies are often associated with feelings of happiness and excitement, while slow tempos and minor melodies can evoke grief and contemplation. The neural basis of music-induced emotions involves multiple brain regions, including the frontal cortex, involved in cognitive processing and emotional regulation; the amygdala, linked to emotional memory and fear responses; and the hippocampus, significant for the formation and retrieval of emotional memories. In addition to neuroimaging evidence, psychophysiological research has shown that music can elicit distinct emotional response patterns measurable through physiological indicators, further supporting the close relationship between musical stimuli and emotional processing (Chen et al., 2018). Together, these findings suggest that multiple neural and physiological systems work in concert to shape the emotional experiences elicited by music (Trost et al., 2024; Zaatar et al., 2023).

Building on Plutchik’s Emotion wheel, the present study focuses on four representative emotions, namely rage, terror, grief, and ecstasy, selected to systematically capture variation along the core affective dimensions of valence (positive vs. negative) and arousal (high vs. low). Accordingly, emotional expression accuracy refers to the correspondence between the intended and perceived emotion of a musical piece. Specifically, rage and terror represent high-arousal negative emotions, grief represents a low-arousal negative state, and ecstasy represents a high-arousal positive emotion. This selection ensures coverage of key regions within the affective space while maintaining experimental tractability.

Importantly, the remaining four emotions in Plutchik’s primary set (e.g., admiration, amazement, loathing, vigilance) were not included for two main reasons. First, these emotions tend to occupy intermediate or mixed positions within the valence–arousal space, often involving more complex or ambiguous appraisal structures, which may introduce additional variability unrelated to the core research question. Second, prior research suggests that emotional recognition accuracy is more stable and interpretable for prototypical, high-intensity emotions than for more subtle or cognitively mediated affective states (Lang, 2019). Therefore, restricting the analysis to four prototypical emotions allows for a clearer and more theoretically grounded comparison between AI-generated and human-composed music in terms of emotional expressiveness.

Although generative AI has rapidly entered the music field, research on the emotional capabilities of AI music remains limited and largely theoretical. Recent studies have increasingly adopted Plutchik’s Emotion Wheel as a theoretical framework for music emotion recognition and generation. For example, Xu et al. (2021) integrated Plutchik’s emotion model into a multimodal music emotion recognition framework using convolutional neural networks, demonstrating the applicability of discrete-emotion structures in AI-based music analysis. More recently, Ji and Yang (2023) proposed a transformer-based approach for emotion-conditioned music generation and recognition, highlighting the growing interest in emotionally controllable AI music system.

However, most studies have focused on algorithms and generation techniques, while relatively little attention has been paid to listener-based validation of emotional expression accuracy. In particular, few studies have directly compared AI-generated music with human-composed music under controlled experimental conditions using a standardized psychological emotion framework (Canyakan, 2024).

In a systematic literature review, Fernando et al. (2024) examined the methodologies used to assess human emotional responses to AI-composed music, concluding that current evaluation approaches are diverse yet lack unified standards, and that empirical data remain generally scarce. Similarly, Dash and Agres (2024) provided a comprehensive technical overview of AI-based affective music generation systems, noting that while significant progress has been made in generation capabilities, insufficient attention has been paid to the “authenticity” or “accuracy” of generated emotions. The authors emphasized that most existing systems prioritize musical coherence and stylistic mimicry over nuanced emotional expressivity, leaving a critical gap in both evaluation frameworks and model design.

Taken together, these studies reveal a core tension in the field: the rapid iteration of technical capabilities is proceeding without a corresponding development of theoretically grounded, empirically validated methods for assessing emotional expression accuracy (Fernando et al., 2024). This gap highlights the need for research using established psychological frameworks and controlled experimental designs to evaluate emotional expression in AI-generated music. Therefore, the present study extends existing research by adopting Plutchik’s Emotion Wheel as a theoretically grounded evaluation framework and empirically examining emotional expression accuracy through listener-based perception tasks. By directly comparing AI-generated and human-composed music across different emotional categories, this study provides behavioral evidence regarding the emotional expression accuracy and limitations of current generative AI music systems.

### 2.4 Theoretical expansion and research hypotheses

Based on Plutchik’s Wheel of Emotions, this study proposes three hypotheses to examine differences in emotional expression accuracy between AI-generated and human-composed music.

First, according to the principle of emotional polarity in Plutchik’s model, opposing emotion pairs (e.g., joy vs. sadness, anger vs. fear) are psychologically most distinct and therefore should yield high recognition accuracy by listeners. However, current AI generation systems may struggle to encode the acoustic features required to convey such oppositions, particularly those involving abrupt dynamic shifts, rhythmic complexity, and textural contrast. Accordingly, Hypothesis 1 is proposed: For opposing emotion pairs, compositions by human composers exhibit significantly higher emotional expression accuracy than AI-generated music.

Second, Plutchik’s Emotion Wheel conceptualizes emotions as varying not only by category but also by intensity within the same emotional family (e.g., serenity–joy–ecstasy). This structure provides a theoretical basis for examining whether listeners can distinguish different emotional intensity levels within a single emotion category. Because AI-generated music may rely more heavily on generalized statistical patterns, it may be less effective in conveying fine-grained variations in emotional intensity. In the present study, this issue was examined specifically within the ecstasy emotion family. Hypothesis 2 is therefore proposed: Human-composed music will demonstrate significantly higher accuracy than AI-generated music in distinguishing emotional intensity levels within the ecstasy category.

Third, while the present study is grounded in a discrete-emotion framework, it incorporates the arousal dimension as a complementary perspective for distinguishing differences in emotional activation levels. High-arousal emotions (e.g., anger) typically require complex and dynamic acoustic features (fast tempo, wide dynamic range, dissonant harmony), whereas low-arousal emotions such as sadness rely on more stable patterns. Given that AI models may be limited in capturing rapid temporal and textural variation, the performance gap between AI and human composers is expected to be larger for high-arousal emotions. Hypothesis 3 states: The difference in emotional expression accuracy between AI-generated and human-composed music is significantly larger for high-arousal emotions than for low-arousal emotions.

To empirically test these hypotheses, emotional expression accuracy is operationalized as the degree of correspondence between the emotion perceived by listeners and the target emotion pre-assigned to each musical excerpt based on established musicological and psychological criteria. Grounded in the discrete emotion categories of Plutchik’s framework, this definition provides a quantifiable and replicable metric for comparing emotional expression across music sources and emotion types. Emotional responses are measured using a forced-choice categorization task, which generates accuracy scores for systematic comparison between AI-generated and human-composed music under standardized conditions.

Together, these hypotheses and measurement approaches provide the theoretical foundation for evaluating emotional expression accuracy in generative AI music.

This paper builds upon Robert Plutchik’s theory and employs a combination of literature review, surveys, and data analysis to investigate emotional expression accuracy in AI-generated versus human-composed music, with the aim of clarifying both the capabilities and limitations of AI in this creative domain.

## 3 Research methods, materials, tools, and subjects

To examine differences in emotional expression between AI-generated and human-composed music, multiple experiments will be conducted. Proper experimental design and material preparation are essential preludes to these tests.

### 3.1 Research methods

The experiment will focus on instrumental works to negate the influence of lyrics on the temporal processing of emotions (Tay and Ng, 2019; Barradas and Sakka, 2022). To control for familiarity, pieces selected will be as unknown to the participants as possible, minimizing prior emotional associations (Sedikides et al., 2022; Stern and Alberini, 2013). The emotional content of these works will correspond to one of the eight primary emotions identified in Plutchik’s Emotion Wheel.

To operationalize emotional expression in music, this study defines emotional expression accuracy as the degree of correspondence between the emotion intended by the composition (as pre-categorized by the research team based on established musicological and psychological criteria) and the emotion perceived by listeners (Turchet and Pauwels, 2022). For the four target emotions: ecstasy, rage, grief, and terror, each musical excerpt was assigned to a single target emotion category based on prior literature, expert consensus from music graduate students and faculty, and the composer’s expressive intent. A standardized scoring scheme was employed: a response was considered “accurate” if the participant selected the emotion category matching the pre-assigned target emotion for that excerpt. Accuracy rates were calculated as the proportion of participants selecting the correct category within each emotion group.

For the secondary experiment within the ecstasy group, a finer-grained evaluation rubric was applied, requiring participants to select among three intensity levels—“ecstasy” (inner circle), “joy” (middle circle), and “serenity” (outer circle)—based on Plutchik’s concentric intensity model. This rubric enabled both categorical accuracy assessment and intensity differentiation. Participants will listen to randomly played AI-generated and human-composed instrumental pieces without knowing their origins. Post listening, they will be asked to identify the emotion expressed in each piece based on Plutchik’s Emotion Wheel. The accuracy of identified emotions will be quantified, and a chi-square analysis will be used to compare the emotional expression accuracy between the two types of music compositions (Fišer et al., 2025).

### 3.2 Experimental materials

The selection of appropriate musical compositions, whether AI-generated or human-composed, is critical. We have established stringent criteria to ensure that only compositions meeting these conditions are used as experimental materials.

#### 3.2.1 Selection of traditional composers’ works

1. Instrumental focus: Only instrumental music will be used to avoid lyric-induced emotional biases (Brattico et al., 2011).

2. Composer and performance quality: Selections will be limited to pieces performed by widely recognized composers and musicians to control for variability in compositional skills and performance techniques.

3. Emotion categorization: To minimize the impact of different emotion categorization methods used in previous studies, this experiment will focus on the four primary emotions with the strongest contrasts in Plutchik’s Emotion Wheel: ecstasy, rage, grief, and terror. The selection of these four emotions is grounded directly in the structural logic of Plutchik’s model. According to the theory, the eight primary emotions are arranged as four pairs of opposites: ecstasy versus grief, admiration versus loathing, terror versus rage, and amazement versus vigilance. By selecting emotions from two opposing pairs: ecstasy versus grief and rage versus terror, the study maximizes emotional contrast and minimizes perceptual overlap. This approach aligns with the theory’s emphasis on emotional polarity as a means of clarifying categorical distinctions. Moreover, these four emotions have been consistently validated in music psychology research as being reliably conveyed through purely instrumental music, thereby supporting the validity of cross-category comparisons (Cheng et al., 2023; Strauss et al., 2024).

4. The remaining four emotions were excluded to reduce participant fatigue and response ambiguity while maintaining experimental control and statistical power.

5. Duration control: Musical piece duration will be standardized to approximately 30 s to balance the clarity of emotional expression and participant response efficiency. This duration is based on previous studies that show significant variability but optimal emotional engagement (Aubé et al., 2013; Baumgartner et al., 2006).

6. Familiarity control: To counteract the Mere Exposure Effect and Memory Enhancement, selections will include music that is likely unfamiliar to the participants (Palumbo et al., 2021; Hekkert et al., 2013). To verify that the selected traditional works were indeed unfamiliar to the participant population, a pre-test familiarity screening was conducted prior to the main experiment. A separate group of 30 participants drawn from the same target population (education majors without formal music training) were asked to indicate whether they had previously heard each of the candidate traditional pieces. Only pieces identified as “unfamiliar” by at least 90% of the screening participants were retained for the main experiment. This procedure minimized the influence of prior exposure on emotional recognition and preference.

Instrumental pieces from esteemed composers that represent the four targeted emotions will be used. Each piece will be about 30 s long to ensure it conveys a complete musical idea independently. Efforts will be made to ensure these pieces or excerpts are not previously known to the participants (Bonassi et al., 2023).

With the collaboration of graduate students and mentors in music studies, along with a review of relevant literature, a total of sixteen musical works were selected. Works expressing the same emotion have been grouped together and edited into single audio tracks using Logic Pro audio processing software, resulting in four tracks that have been exported in MP3 format.

#### 3.2.2 Selection of generative AI music works

In recent years, with the rapid development of artificial intelligence technology, generative AI music models have become a new force in music creation. These models use deep learning algorithms to generate music in various styles and forms, bringing many conveniences and innovations to the field of music creation (Mycka and Mańdziuk, 2024). Six mainstream AI music generation models were examined and provides a brief overview of each model (Lam, 2024).

So-vits-Svc is an advanced music generation model based on deep learning technology, which has attracted widespread attention in the fields of music composition and audio processing in recent years (Ji et al., 2023). Its main advantage is its ability to produce high-quality audio that is very similar to human singing, exhibiting natural and smooth musical characteristics. In addition, So-vits-Svc supports the generation of various styles and types of music, allowing users to flexibly adjust parameters as needed, making it suitable for applications such as game music and background music. This flexibility and versatility make it extremely practical during the creative process. In addition, So-vits-Svc can generate music in a short time, significantly improving creative efficiency, especially for commercial projects that require rapid iteration. Its user-friendly interface and tools make it easy for users even without professional music background to participate in composition. However, So-vits Svc also has some disadvantages.

While the generated music is of high quality, it often lacks the uniqueness and emotional depth characteristic of human compositions, which can result in insufficient artistic value. Furthermore, the performance of the model depends heavily on the quality and diversity of the training data; if the training data is not rich or contains biases, the generated music may not meet the user’s expectations. And So-vits-Svc requires extensive computing resources during training and generation, which may burden smaller teams or individual users. Finally, using generative models for music creation may raise copyright issues, and ensuring the legality of generated works remains a concern. Therefore, although So-vits-Svc is a high-quality and flexible music generation tool, it lacks uniqueness and emotional depth and is not suitable as experimental material for this study.

The OpenAI MuseNet model is a large-scale deep learning model that can simulate various music styles and 10 different instruments to create new musical works up to 4 min long. This reflects its main strengths: strong creativity and imitation capabilities. By learning the commonalities and differences of various musical styles, it deepens the understanding of musical styles and instrumental sounds to serve the creation of new music. However, because the music generated by this model comes from a machine learning algorithm rather than a creative process, the final product may lack real emotional and innovative depth (Zhang, 2025). Furthermore, the reliance on large amounts of training data poses significant challenges to building completely fair and unbiased datasets. Because music perception is influenced by cultural and subjective factors, large datasets alone cannot guarantee universal applicability across different populations. Additionally, the use of MuseNet raises legal issues related to copyright. While the resulting work is original, the training process involves large amounts of existing music, which may infringe the rights of composers and copyright owners. In addition, OpenAI MuseNet has not released a new version in several years, and its music generation quality does not exceed other generative music models. Therefore, MuseNet is considered unsuitable as experimental material for this study.

Google Music LM: Developed by Google, Music LM represents an innovative leap in music generation, utilizing advanced deep learning technology to create music segments from user-provided text prompts. This tool offers substantial benefits in terms of innovation and scalability, making it particularly accessible for non-musicians who wish to explore music creation. Music LM is characterized by its universality and ease of use, allowing users to input simple text descriptions and receive rich, varied musical outputs. This significantly reduces barriers to music creation and opens new avenues for music education and entertainment. However, Music LM faces challenges, particularly when processing extremely complex or nuanced musical arrangements, and the audio quality may not consistently meet professional standards. Despite its user-friendly nature, the relative lower quality of the generated music deems Music LM unsuitable as experimental material for this study.

Udio Music Generation Model: Udio is a robust AI music creation tool designed to enhance both the efficiency and creativity of music production. It supports the generation of music across various styles, catering to diverse user needs. Key advantages of Udio include its high-quality sound effects and detailed musical compositions that achieve a professional level of depth and layering. The model offers extensive music control options, allowing for significant customization and personalization in music creation. Despite its strengths, Udio tends to produce compositions with a high degree of repetitiveness, and the abrupt endings of its musical pieces often lead to unsatisfactory completions. These factors suggest that Udio is better suited for tasks in audio processing and sound design rather than as experimental material for this study.

Meta Music Gen: Meta Music Gen is an advanced music generation model developed by Meta, designed to foster the creation of innovative and engaging musical works through artificial intelligence. By integrating deep learning algorithms with music theory, Meta Music Gen is capable of automatically generating music in various styles and rhythms, adapting to different user contexts. The model’s primary strength lies in its rapid generation of diverse music, learning from a vast corpus of musical data to capture the essence of various styles. It is user-friendly, enabling individuals without extensive musical background to engage with the tool easily, thus lowering the barriers to music creation. However, Meta Music Gen has notable limitations. Although it can produce music in a range of styles, the precision of its music generation often falls short, with significant discrepancies evident between the generated compositions and the descriptive prompts. Additionally, the music generated is frequently repetitive, with multiple pieces from the same prompt showing considerable homogeneity. These issues render Meta Music Gen unsuitable as experimental material for this study.

Suno AI: Suno AI is a generative AI music model developed by a U.S. startup, exemplifying an advanced artificial intelligence product that harnesses the latest AI technologies to convert simple text prompts into rich and complex audio outputs. Notably, the update frequency for Suno AI is relatively high, with its version V3.5 released in 2024, offering a broad spectrum of creative options. This model allows users to freely select elements such as music style, rhythm, and tonality, facilitating the creation of diverse music that caters to various creative demands. This significantly expands the possibilities within the music industry. Moreover, the music generation process of Suno AI is based on real-time calculations, offering considerable advantages in terms of time and cost efficiencies. Unlike traditional music creation processes that often require significant time and effort, Suno AI enables users to swiftly produce music at a professional level, thereby enhancing creative productivity and reducing production costs (Wang and Gong, 2025). Suno AI also impacts globally, providing sharing features that allow users to easily disseminate their works, thereby attracting more listeners and enhancing communication and collaboration among music creators.

Suno AI is built upon a transformer-based architecture similar to that used in large-scale language models but adapted for audio tokenization and synthesis (Athreya and Dash, 2025). Unlike traditional symbolic music generation systems that operate on MIDI or score-based representations, Suno AI employs an end-to-end audio generation pipeline that converts text prompts directly into waveform audio through a latent diffusion process conditioned on textual and musical embeddings. This architecture enables real-time generation and flexible style control but also imposes constraints on the model’s ability to encode fine-grained emotional dynamics. Emotion expression in Suno AI is primarily driven by prompt-level semantic conditioning rather than explicit emotion modeling at the structural or performance level. The model maps descriptive emotional keywords (e.g., “rage,” “ecstasy”) to latent representations learned from training data, yet it lacks explicit mechanisms to control temporal dynamics, articulation, or micro-expressive variations that are critical for conveying complex or intense emotions. As a result, although Suno AI can generate music in broad stylistic categories, it may still struggle to convey nuanced or high-arousal emotions. While it has certain limitations in encoding fine-grained emotional dynamics, these very limitations make it a suitable choice for investigating the current capabilities and boundaries of state-of-the-art text-to-audio generation. Therefore, despite its limitations, Suno AI remains a suitable model for evaluating emotional expression in contemporary generative music systems.

To enhance the rigor of model selection, the present study adopted a multi-criteria evaluation framework to compare candidate generative AI music systems. Specifically, five key dimensions were defined based on prior literature on music perception and AI evaluation: (1) audio quality, (2) emotional controllability, (3) structural coherence, (4) generation stability, and (5) ecological validity (Hariyady et al., 2024). Each model was evaluated using a 5-point rating scale (1 = very low, 5 = very high) based on pilot listening tests conducted by three independent raters with basic music perception training. The final score for each model was calculated as the mean across dimensions.

As shown in Table 1, Suno AI V3 achieved the highest overall score, particularly in audio quality and structural coherence, ensuring that the generated stimuli meet the perceptual realism required for experimental tasks. Therefore, Suno AI V3 was selected as the stimulus generation model.

**TABLE 1:** Quantitative evaluation of candidate AI music models.

Model	Audio quality	Emotional control	Structural coherence	Stability	Ecological validity	Mean score
So-vits-Svce	3.67	3.00	3.33	4.00	3.33	3.46
MuseNet	4.00	2.67	3.67	3.67	3.33	3.47
MusicLM	3.67	2.67	3.33	3.33	3.67	3.33
Udio	4.67	3.33	4.00	4.00	3.33	3.87
MusicGen	4.00	3.00	3.67	3.67	3.67	3.60
Suno AI V3	4.67	3.67	4.67	4.0	4.0	4.07

To ensure transparency and replicability, all stimuli were generated using Suno AI V3 under the platform’s default generation settings. The Suno AI web platform does not provide user-level access to parameters such as temperature or random seed. Therefore, all generations were conducted using the default system configuration to maintain consistency across stimuli. All musical excerpts were generated as instrumental orchestral-style pieces with a fixed duration of 30 s. The instrumentation was restricted to a consistent orchestral palette (strings, brass, and woodwinds), and no lyrics or vocal elements were included to avoid semantic interference. The prompt template consisted of four components: (1) emotional descriptor, (2) tempo range, (3) tonal mode, and (4) instrumentation. Among these, emotion was the primary independent variable, whereas tempo and tonal mode were varied in a theoretically controlled manner based on established associations in music psychology.

Specifically, high-arousal emotions (rage, ecstasy) were generated with faster tempo ranges, whereas low-arousal emotion (grief) was generated with slower tempo. Negative-valence emotions (rage, terror, grief) were generated in minor mode, while positive-valence emotion (ecstasy) was generated in major mode. Aside from these theoretically grounded adjustments, all other parameters remained consistent across conditions.

The standardized prompt template was as follows: “An instrumental orchestral piece expressing [TARGET EMOTION], with a [TEMPO RANGE] tempo, in [TONAL MODE], using consistent orchestral instrumentation. The music should clearly convey the emotional quality of [TARGET EMOTION] while maintaining structural coherence.”

Using this template, 20 unique compositions were generated for each emotion condition via Suno AI V3 under default settings, resulting in a total of 80 candidate stimuli (20 × 4 emotions).

Following generation, all stimuli were screened to exclude excerpts with extreme deviations in tempo, instrumentation, or structural irregularities, ensuring comparability across conditions. This procedure enhances internal validity by isolating emotional intent as the primary manipulated variable.

A rigorous selection process was employed to narrow the 80 generated pieces to the final 16 (four per emotion group). This process involved three stages: (1) Technical quality screening that pieces with obvious artifacts, abrupt endings, or poor audio fidelity were excluded. (2) Emotional consistency evaluation, a panel of three graduate students in music studies, who were blinded to the study’s hypotheses, independently rated each piece for the presence and dominance of the target emotion using a 5-point Likert scale. Only pieces with a mean rating of ≥ 4.0 for the target emotion and no secondary emotion rated above 3.0 were retained. (3) Final selection, from the pool of pieces meeting the above criteria, four pieces per emotion were selected to match, as closely as possible, the average duration and structural complexity of the traditional composer work used in the study. This process ensured that the selected AI-generated pieces were not only of high technical quality but also predominantly expressed the intended target emotion without significant emotional ambiguity (Eerola and Saari, 2025).

To validate that the selected AI-generated pieces expressed only the target emotions and did not inadvertently convey unintended affective content, a pre-test validation study was conducted with a separate group of 30 preliminary experiment participants (drawn from the same population but not involved in the main experiment). These participants listened to each selected AI-generated piece and selected the primary emotion they perceived using the full eight-category emotion wheel. For all 16 pieces, the target emotion was selected by at least 80% of participants, and no piece received more than 10% endorsement for any non-target emotion (Korsmit et al., 2023). This preliminary experiment confirmed that the selected AI-generated stimuli reliably conveyed the intended target emotions with minimal cross-emotional confusion.

Following the guidelines on the Suno AI official website, I generated eighty pieces of music using prompts designed to evoke specific emotions: intense rage, fear, joy, and grief. Through the above-described selection and validation procedures, sixteen pieces were chosen to represent the AI-generated music for this study. These works, each expressing a specific emotion, were grouped together. Using Logic Pro X audio processing software, the music from each group was edited and merged into a single audio track, resulting in a total of four tracks that were exported in MP3 format. Further details of these selections are provided in Table 2.

**TABLE 2:** Experimental music materials.

Musical mood	Source	Group name	Song title	Composer	Duration(s)
Joy	Human	H1 A1	Wedding day at Troldhaugen	Edvard Grieg	30
	Human		Sunrise string quartet, Op.76, No.4	Joseph Haydn	30
	Human		The four seasons: spring	Antonio Vivaldi	30
	Human		Symphony No. 7 in a major, Op. 92, III. presto	Beethoven	30
	AI		Musical work a1	Suno AI	30
	AI		Musical work a2	Suno AI	30
	AI		Musical work a3	Suno AI	30
	AI		Musical work a4	Suno AI	30
Rage	Human	H2 A2	Étude, Op. 25, No. 11.	Chopin	30
	Human		Revolutionary etude Op. 10, No. 12	Chopin	30
	Human		Prelude in G minor, Op. 23, No. 5	Sergei Rachmaninoff	30
	Human		Symphonie fantastique, Op. 14, March to the scaffold	Hector Berlioz	30
	AI		Musical work b1	Suno AI	30
	AI		Musical work b2	Suno AI	30
	AI		Musical work b3	Suno AI	30
	AI		Musical work b4	Suno AI	30
Grief	Human	H3 A3	Piano Sonata No.12 in A-flat major Op.26	Beethoven	30
	Human		Les Contes d’ Hoffmann	Jacques Offenbach	30
	Human		Funeral March Op. 72, No. 2	Chopin	30
	Human		Ballade No.1, Op.23	Franz Liszt	30
	AI		Musical work c1	Suno AI	30
	AI		Musical work c2	Suno AI	30
	AI		Musical Work c3	Suno AI	30
	AI		Musical work c4	Suno AI	30
Terror	Human	H4 A4	Lou is dead	Alan Silvestri	30
	Human		Psycho suite	Bernard Herrmann	30
	Human		Night on bald mountain	Modest Mussorgsky	30
	Human		Dante symphony	Boris Tchaikovsky	30
	AI		Musical work d1	Suno AI	30
	AI		Musical work d2	Suno AI	30
	AI		Musical work d3	Suno AI	30
	AI		Musical work d4	Suno AI	30

### 3.3 Experimental questionnaire and subjects

The experimental music pieces were classified into four basic emotions: ecstasy, rage, grief, and terror. These were further divided based on their origin—traditional composers or AI-generated—resulting in eight distinct musical groups. A cohort of 283 education majors from a higher vocational college in Sichuan Province, China, participated in the study. These participants, none of whom had received professional music training, were on average 20.5 years old. The experimental sessions were conducted in a controlled, quiet classroom setting and lasted about 20 min.

#### 3.3.1 Experimental questionnaire design

The questionnaire was administered in paper format and consisted of two main sections. The first section collected basic demographic information (age, gender, and prior music training status) to confirm eligibility and characterize the sample. The second section presented the experimental listening task. For each musical excerpt, participants were instructed to select, from a list of the eight primary emotion categories from Plutchik’s Emotion Wheel (ecstasy, admiration, terror, amazement, grief, loathing, rage, vigilance), the single emotion they perceived as most strongly expressed in the piece. A forced-choice format was used, requiring participants to select exactly one category per excerpt. This design ensured that accuracy could be quantified unambiguously by comparing each participant’s selection against the pre-determined target emotion for that excerpt. No partial credit was assigned for semantically related emotions (e.g., selecting “loathing” for a “rage” target), as the focus was on discrete emotion recognition accuracy.

For the follow-up experiment conducted within the ecstasy group, the response format was modified to include the three intensity levels (ecstasy, joy, serenity) based on Plutchik’s concentric circles, allowing for finer-grained assessment of emotional intensity recognition. The questionnaire included clear written instructions and a brief illustration of the Emotion Wheel to ensure participants understood the task.

The musical pieces were arranged into groups corresponding to the four fundamental emotions delineated by Plutchik’s Emotion Wheel: ecstasy, rage, grief, and terror. Participants were not informed of the origins of the music pieces, whether AI-generated or traditionally composed. The eight musical groups (four emotions × two sources) were presented in a fully randomized order across participants to control for order effects. Within each group, four musical excerpts were played sequentially, with a fixed inter-stimulus interval of 3 s between excerpts to allow for perceptual separation without disrupting the listening experience. All audio playback was conducted using JBL GO3 Bluetooth speakers with the volume fixed at a constant level of 65 dB (measured at the participant’s listening position) to ensure uniform auditory presentation across all sessions. Music groups were played randomly, and participants were tasked with selecting the “emotion group” they believed each piece represented, based on the Emotion Wheel.

#### 3.3.2 Experimental subjects and setting

Participants were recruited from a cohort of students majoring in education at a higher vocational college in Sichuan Province, China. After data screening, a total of 283 valid participants were included in the final analysis. Participant demographic characteristics are presented in Table 3. The sample consisted of 130 males (45.9%) and 153 females (54.1%), with a mean age of 20.5 years (SD = 0.8). All participants were education majors. The total duration of the experimental session was approximately 20 min. All experimental sessions were conducted in a dedicated quiet classroom with ambient noise levels below 40 dB. Participants were seated at individual desks with a minimum spacing of 1.5 m to prevent interaction during the listening task. The JBL GO3 Bluetooth speakers were positioned centrally in the room to ensure consistent sound distribution. Each excerpt was played in full, followed by a 5-s response window before the next excerpt began. A brief practice trial was conducted prior to the formal experiment to familiarize participants with the response format and listening procedure, using musical examples not included in the main experimental materials. All participants provided written informed consent prior to participation, indicating that they had fully understood the purpose and procedures of the study and voluntarily agreed to take part. The research team adhered strictly to established standards of data confidentiality and privacy protection, ensuring that participants’ personal information was not disclosed. All data were processed using anonymization procedures.

**TABLE 3:** Participant characteristics.

Variable	Category	*N*	%
Gender	Male	130	45.9
	Female	153	54.1
Age	Mean (SD)	20.5 (0.8)	−
Major	Education	283	100

The use of written informed consent in this study demonstrates adherence to core ethical principles, particularly respect for individual autonomy and voluntary participation. From a methodological standpoint, informed consent serves as an essential procedural safeguard to ensure transparency, participant understanding, and voluntariness, even in studies that qualify for exemption from formal ethical review.

This study employed standardized self-report questionnaires to collect attitudinal and perceptual data. No experimental manipulation, behavioral intervention, deception, or exposure to risk-inducing stimuli was involved. In accordance with widely accepted methodological standards in the social sciences, survey-based research of this type is considered minimal risk, as it does not alter participants’ behavior, health status, or social conditions.

## 4 Experimental results and data analysis

Following comprehensive preparations, we conducted experiments involving 300 participants and performed detailed statistical analyses on the collected data.

Table 4 summarizes the distribution of emotion responses and recognition accuracy across conditions. Compared with human-composed music, AI-generated music generally showed lower recognition accuracy and broader distributions of non-target emotional responses, particularly in the rage, ecstasy, and terror conditions. The largest difference in recognition accuracy was observed for rage (78.40% vs. 53.00%), followed by ecstasy (63.60% vs. 42.10%). In contrast, grief showed relatively high recognition accuracy in both conditions.

**TABLE 4:** Distribution of emotion responses and recognition accuracy (%).

Emotion target	Source	Correct response	Main misclassification(s)
Rage	Human	78.40	Terror (14.50)
Rage	AI	53.00	Terror (19.10), Loathing (13.40)
Ecstasy	Human	63.60	Admiration (18.40)
Ecstasy	AI	42.10	Vigilance (23.30), Amazement (18.70)
Grief	Human	92.20	Loathing (4.90)
Grief	AI	88.00	Loathing (6.70)
Terror	Human	63.60	Rage (19.80)
Terror	AI	46.60	Rage (19.40), Amazement (14.50)

To examine whether emotion recognition accuracy differed between human-composed and AI-generated music across emotional categories, chi-square analyses were conducted. As shown in Table 5, significant associations between music source and recognition accuracy were observed for rage (χ^2^ = 40.66, *p* < 0.001, *V* = 0.27), ecstasy (χ^2^ = 25.53, *p* < 0.001, *V* = 0.21), and terror (χ^2^ = 16.46, *p* < 0.001, *V* = 0.17). According to Cohen’s (1988) guidelines, Cramer’s V values of 0.10, 0.30, and 0.50 indicate small, moderate, and large effect sizes, respectively. Therefore, the observed effects ranged from small to moderate in magnitude. In contrast, the difference for grief was not statistically significant (χ^2^ = 2.44, *p* = 0.12, *V* = 0.07).

**TABLE 5:** Chi-square test results.

Emotion	χ^2^	*p*	Cramer’s V
Rage	40.66	< 0.001	0.27
Ecstasy	25.53	< 0.001	0.21
Grief	2.44	0.12	0.07
Terror	16.46	< 0.001	0.17

To further examine whether the effect of music source on emotion recognition accuracy differed across emotional categories, a hierarchical logistic regression analysis was conducted. Emotion type was entered as a categorical variable with four levels (ecstasy, rage, terror, and grief), with grief specified as the reference category. Music source was dummy-coded as 0 = human-composed music and 1 = AI-generated music. As shown in Table 6, the results of Step 1 indicated that both music source and emotion type significantly predicted recognition accuracy (*R*^2^ = 0.13). Specifically, AI-generated music showed significantly lower recognition accuracy than human-composed music (*B* = −0.85, *SE* = 0.10, *p* < 0.001). Compared with grief, ecstasy (*B* = −2.18, *SE* = 0.17, *p* < 0.001), rage (*B* = −1.62, *SE* = 0.17, *p* < 0.001), and terror (*B* = −2.09, *SE* = 0.17, *p* < 0.001) exhibited significantly lower recognition accuracy.

**TABLE 6:** Hierarchical logistic regression predicting emotion recognition.

Variables	Step 1			Step 2		
	B	*SE*	Exp	B	*SE*	Exp
Source	−0.85***	0.10	0.43	−0.45	0.29	0.64
Emotion (1)	−2.18***	0.17	0.11	−1.50**	0.55	0.22
Emotion (2)	−1.62***	0.17	0.20	−0.46	0.57	0.63
Emotion (3)	−2.09***	0.17	0.12	−1.67**	0.55	0.19
Emotion (1) * source				−0.42	0.34	0.66
Emotion (2) * source				−0.72*	0.34	0.49
Emotion (3) * source				−0.24	0.34	0.78
Constant	3.58	0.22	35.73	2.92***	0.34	18.58
R2	0.13			0.14		

*p < 0.05; **p < 0.01; ***p < 0.001.

To test the potential moderating role of emotion type, interaction terms between music source and emotion category were added in Step 2. As shown in Table 6, the explained variance increased only slightly (Δ*R*^2^ = 0.01, total *R*^2^ = 0.14). After including the interaction terms, the main effect of music source was no longer significant (*B* = −0.45, *SE* = 0.29, *p* = 0.121). Ecstasy (*B* = −1.50, *SE* = 0.55, *p* = 0.007) and terror (*B* = −1.67, *SE* = 0.55, *p* = 0.003) remained significantly lower than grief, whereas the difference between rage and grief was no longer significant (*B* = −0.46, *SE* = 0.57, *p* = 0.424). Among the interaction effects, only the interaction between rage and music source reached statistical significance (*B* = −0.72, *SE* = 0.34, *p* = 0.036), indicating that the difference between AI-generated and human-composed music was particularly pronounced for rage-related emotional expression. Overall, the interaction effects were relatively weak, suggesting that emotion type showed only limited moderating effects on the relationship between music source and emotion recognition accuracy.

A chi-square analysis revealed a significant difference in intensity-level recognition between AI-generated and human-composed music within the ecstasy emotion family, χ^2^ = 71.73, *p* < 0.001, Cramer’s *V* = 0.36. The distribution of participants’ emotional intensity classifications is presented in Table 7. Compared with human-composed music, AI-generated music was substantially less likely to be identified as ecstasy and more likely to be categorized as lower-intensity emotional states such as joy or serenity.

**TABLE 7:** Emotional intensity classification within the ecstasy group (%).

Emotional intensity	Human (%)	AI (%)
Ecstasy	58.3	45.2
Joy	40.3	29.0
Serenity	1.4	25.8

## 5 Conclusion

This study examined differences in emotional expression accuracy between music generated by Suno AI and human-composed works. The findings indicate that the effect of music source on emotion recognition is not uniform across emotional categories, but varies depending on the type of emotion.

Specifically, chi-square analyses revealed significant differences between human and AI-generated music in the rage, ecstasy, and terror conditions (*p* < 0.001), whereas no significant difference was observed for grief (χ^2^ = 2.44, *p* = 0.119). This pattern suggests that the advantage of human-composed music may vary across different emotional categories. In particular, both human-composed and AI-generated music showed relatively high and comparable recognition accuracy for grief, which implies that low-arousal emotions may be more readily captured by current AI music generation systems.

In contrast, substantial differences were observed for high-arousal emotions such as rage and ecstasy, where human-composed music consistently yielded higher recognition accuracy. Terror showed a moderate gap, suggesting an intermediate level of difficulty. These findings suggest that emotional expression accuracy may be related to differences in emotional arousal and complexity. High-arousal emotions often involve relatively rapid temporal changes, greater dynamic contrast, and more complex structural patterns, which may pose additional challenges for current AI systems in terms of reproduction (Gomez and Danuser, 2007). The logistic regression results further support this interpretation. While the main effect of emotion was significant, the interaction between emotion and source was not significant overall, indicating that the influence of music source does not differ dramatically across all emotion categories. However, the pattern of coefficients suggests that differences between human and AI music are more pronounced in specific emotions.

Although the present study did not directly examine neural mechanisms, previous neuroscience research has shown that music-evoked emotions involve brain regions such as the amygdala, striatum, and prefrontal cortex (Koelsch, 2014). These findings may provide a possible framework for understanding why differences emerged between AI-generated and human-composed music, particularly for high-arousal emotions. However, the current behavioral data do not allow direct conclusions regarding the underlying neural processes. Future studies combining behavioral paradigms with EEG or neuroimaging techniques could further investigate the neural mechanisms associated with emotional responses to AI-generated music.

Several actionable implications emerge from these findings. First, improving emotional expressivity in AI-generated music may require incorporating explicit representations of emotional dimensions into the generation process. Second, training datasets should be curated to include a more balanced distribution of emotional categories, particularly high-arousal states that appear underrepresented. Third, model architectures may benefit from enhanced temporal control mechanisms to better capture dynamic emotional transitions over time. Overall, the results suggest that while current generative AI systems can approximate certain emotional expressions, they remain limited in conveying high-intensity, dynamically complex emotions.

## 6 Prospects and limitations

This research evaluated the accuracy of emotional expression in generative AI music, with a particular focus on contrasting AI-generated works with those of traditional composers based on the “Emotion Wheel” theory. Detailed experimental analysis indicated that despite AI’s substantial progress in mimicking various musical styles and technical processes, it falls short in capturing profound emotional expressions, particularly in terms of complex and subtle emotional nuances, compared to traditional music compositions. Future research could address these gaps by:

Enhancing AI’s Understanding of Complex Emotions: Future research could focus on developing emotion-conditional generative models that explicitly encode Plutchik’s dimensional structure—such as valence, arousal, and intensity, as conditioning variables during the generation process. Concretely, this may involve constructing labeled datasets with fine-grained emotional annotations (e.g., using continuous valence–arousal ratings or intensity tiers from the Emotion Wheel) and training transformer-based or diffusion-based architectures with multi-task loss functions that jointly optimize for musical coherence and emotional discriminability. Researchers could also explore reinforcement learning from human feedback (RLHF) to fine-tune models toward higher emotional expression accuracy, using listener accuracy rates as a reward signal.

Exploring Collaborative Work Models: Rather than treating AI and human composers as mutually exclusive sources, future research could design and empirically evaluate interactive co-creation frameworks. For instance, human composers could provide high-level emotional structures or expressive intentions (e.g., dynamic contours, intensity trajectories), while AI systems generate complementary musical layers in real time. Alternatively, AI could generate multiple variants of a musical passage, from which human composers select, refine, and re-integrate into a larger emotional arc. Such hybrid workflows could be tested through controlled experiments comparing listener emotional responses to purely human, purely AI, and collaboratively created works, thereby assessing whether collaboration enhances emotional accuracy beyond either approach alone.

Expanding Practical Application Scenarios: Future research could move beyond laboratory-based listening tasks by deploying AI-generated music in real-world contexts such as film scoring, game sound design, and music therapy. In film music, for example, researchers could systematically evaluate whether AI-generated soundtracks achieve comparable emotional alignment with narrative scenes as human-composed scores, using both audience ratings and physiological measures (e.g., skin conductance, heart rate variability). In music therapy, AI systems could be designed to adapt musical output in real time based on patient emotional responses, with clinical trials assessing efficacy for conditions such as anxiety or depression. These applied studies would not only test the generalizability of current findings but also inform iterative model improvements grounded in domain-specific requirements.

While this study provides empirical evidence regarding the comparative emotional accuracy of AI-generated and human-composed music, several critical limitations must be acknowledged beyond sample selection and experimental design. First, the study does not address the neural mechanisms underlying music-induced emotional perception. Music-evoked emotions involve complex brain networks, including the limbic system (amygdala, hippocampus), prefrontal cortex, and reward circuits (striatum), which mediate emotional appraisal, memory integration, and aesthetic pleasure. Without neurophysiological measurements (e.g., fMRI, EEG), the current findings remain at the behavioral level and cannot elucidate whether AI-generated and human-composed music engage these neural substrates differently. Future research should integrate neuroimaging techniques to examine whether the observed differences in emotional accuracy correspond to distinct patterns of neural activation.

Second, the study does not analyze emotional biases embedded in the training data of AI music models. Suno AI, like most generative models, is trained on large-scale datasets that may overrepresent certain musical genres, cultural traditions, and emotional expressions while underrepresenting others. For instance, high-arousal negative emotions such as rage may be statistically underrepresented in commercially available music corpora compared to mid-tempo, emotionally neutral content. This dataset bias likely contributes to the model’s lower accuracy in generating high-intensity emotions. Future research should conduct systematic audits of training corpora to identify and mitigate such biases, potentially through curated datasets that ensure balanced emotional representation.

Third, the study does not explore variations in emotional expression across different music genres. The selected traditional works span classical and romantic periods, while AI-generated pieces were prompted without genre-specific constraints. It remains unclear whether AI’s emotional accuracy varies by genre (e.g., classical vs. jazz vs. electronic) and whether genre-specific training or conditioning could improve performance. Future studies should systematically compare AI-generated music across multiple genres to assess whether emotional expression accuracy is moderated by stylistic factors.

Fourth, the study relies on a forced-choice emotion categorization task based on Plutchik’s discrete emotion model, which may not capture the dimensional nature of emotional experience (e.g., valence-arousal space) or the possibility of mixed emotions. Listeners may perceive multiple emotions simultaneously or experience emotions that do not map neatly onto discrete categories. Future research should employ both categorical and dimensional measures, as well as continuous response methods, to capture the full complexity of emotional responses to AI-generated music.

Fifth, the present study is limited by the homogeneity of the sample. All participants were recruited from a single vocational college in Sichuan Province, China, and were all education majors with no formal musical training. This relatively uniform cultural background and academic profile may restrict the generalizability of the findings. In particular, variations in musical expertise, cultural exposure, and aesthetic experience were not systematically examined in the current study. Therefore, caution is warranted when generalizing the results to broader populations, such as individuals with professional music training or participants from different cultural contexts. Future research should include more diverse samples across educational backgrounds, cultural settings, and levels of musical expertise to enhance external validity.

Finally, the study focuses exclusively on instrumental music to control for lyrical confounds, but this limits generalizability to vocal music, where lyrics play a significant role in emotional communication. Future research should examine whether AI-generated vocal music exhibits similar or different patterns of emotional accuracy, particularly given that lyrical content may provide additional semantic cues that could enhance or interfere with emotional expression.

Continuous exploration and discussion of these prospects and limitations will enable future research to provide a more comprehensive understanding of the potential and challenges of generative AI music, offering deeper insights into the integration of musical art and technology.

## Data Availability

The raw data supporting the conclusions of this article will be made available by the authors, without undue reservation.
